# Selection of Indicator Bird Species as a Baseline for Knowledge Assessment in Biodiversity Survey Studies

**DOI:** 10.3390/ani13132230

**Published:** 2023-07-06

**Authors:** Talia Härtel, Janina Vanhöfen, Christoph Randler

**Affiliations:** Department of Biology, Eberhard-Karls-University Tübingen, Auf der Morgenstelle 24, 72076 Tuebingen, Germany; janina.vanhoefen@uni-tuebingen.de (J.V.); christoph.randler@uni-tuebingen.de (C.R.)

**Keywords:** knowledge about species, expert rating, bird species, citizen science, knowledge survey

## Abstract

**Simple Summary:**

Knowledge about species has been surveyed many times in research in the past. To our knowledge, species selection has never been properly justified and there is no consensus on which species should be used as a baseline for knowledge about species in the public. Based on database analysis and two expert panel studies, a list of 50 bird species (occurring in Germany) is provided at the end of this paper. The list can be used by educational institutions, for example, but also for research to make studies about knowledge about species more comparable in the future.

**Abstract:**

The loss of Earth’s biodiversity is accompanied by a loss of public knowledge about species. Many scientists are convinced that knowledge about species is an important prerequisite to interest and investment in species conservation. In the past, knowledge about species has mostly been assessed using birds, but there is no consensus on which birds could serve as a baseline for knowledge about species in the general public. The aim of this study is to provide a list of the ‘golden 50′ bird species in Germany that can be used by educational institutions, as well as studies about species knowledge to make them more comparable. The list can also serve as a basis for the selection of so-called flagship species, which are used for the protection of habitats and other species due to their high likeability. To achieve this, three consecutive steps were conducted: an analysis of bird-related databases to determine which species might be common and known and two expert panel studies. The data analysis included several factors: species characteristics, Citizen Science data, public value and importance, and scientific studies. In both the first and second rounds, experts were asked for their opinion on which species should be known by the general public in Germany. The first expert panel, which consisted of only a small group of experts (n = 6), was mainly used to reduce the number of species for the second panel. In the second expert panel, 197 ornithologically qualified experts from all over Germany were asked for their assessment. The correlations between the expert assessments and the different variables were all significant (except for the species trait “colourfulness”), which validates the selection process used here and consequently the species list that has been compiled. The selection process can also be applied to other biogeographical regions or taxa.

## 1. Introduction

Recent decline in bird populations is one of the many components of the biodiversity crisis [1]. The latter is particularly caused by humans, as natural resources are overexploited [2], habitats are destroyed and fragmented [3], and human–wildlife conflicts are arising [4]. Together with climate change, this will most likely accelerate species extinction in the future [5]. The decline in biodiversity is accompanied by a decline in knowledge about avian species identification [6] as well as deeper knowledge about ecology and natural history (e.g., about their habitat [7]). This, in turn, is linked to a loss of the experience of nature, for example, listening to birdsong [8]. We are thus faced with a triad of declines, in bird species, knowledge of birds, and experience with them.

Knowledge about species, understood as the identification and naming of species [9], is important from many perspectives. For one, knowledge of species strengthens the connection to nature and counteracts the currently declining perception of natural elements (so-called plant/nature blindness) [10,11]. A lack of knowledge about species also makes it difficult to understand the systematics of organisms [12]. In addition, knowledge of species is important for a deeper understanding of ecology and serves as a starting point for an understanding of biodiversity [13,14]. The importance of knowledge about species for nature conservation and environmental protection is particularly relevant in regard to the biodiversity crisis. Knowledge about species is an important prerequisite for the protection of biodiversity [15], and high knowledge about species also implies higher environmental awareness [16]. People with a high level of knowledge about species also have more positive attitudes towards animals [17], which is important when it comes to protecting certain animal species [18]. Due to the decline in bird populations, their conservation is extremely important [1]. Birds serve as indicators of the biodiversity of an ecosystem [19], and they also provide some important ecosystem services including the eating of pests, the pollinating of flowers, and the dispersal of seeds [20]. Thus, knowledge of bird species is important for bird conservation.

Knowledge about bird species must therefore be fostered in the future, regardless of formal education or informal learning settings. Many studies on knowledge about bird species have been carried out in recent years, e.g., [21,22], although there are some precursors in earlier times [9,23]. Yet, the species surveyed in these studies are often not uniform and there is no consensus on which bird species a person should be able to identify. This makes the studies difficult to compare and strengthens the need for a baseline of bird species to improve them. Hence, it is important to find out which species can serve as a baseline for knowledge about species and as representatives of species diversity. The curricula of educational institutions could then be adapted to this. In addition, a baseline of species might help to record changes in knowledge about species over time, thus making studies on knowledge about species more comparable. These studies are also very important because they often link knowledge about species with personal factors, e.g., interests and attitudes [10,24], and thus address the question of the best way to promote knowledge about species. Furthermore, such a baseline of bird species could also be used for selecting so-called ‘flagship species’ that are important for conservation purposes [25].

Previous studies on knowledge based their bird species selection on a variety of different criteria, i.e., population size [6,9,10], garden bird counts, Citizen Science (CS) data [7,21,26], breeding bird surveys, or presence in school textbooks [24,27]. However, in most studies on knowledge, the choice of species was based on only one or a few criteria. Thus, there is a lack of agreement and consensus about the species selection itself and even a lack of agreement about the procedure of species selection. To establish a baseline of species that can be used for studies or educational purposes, it is important to refer to a variety of criteria in order to induce as little bias in the selection as possible and adequately represent bird biodiversity.

The shortcoming when focusing only on population size, for example, is that population size is negatively related to body size [28], and selecting this as an indicator will result in the selection of smaller species because there are many more individuals of a smaller species in a given geographic range. Furthermore, population size is influenced by phylogeny. In the example of parids (family: *Paridae*), all species in Germany are closely related, have a similar size (due to phylogenetic relationship), and three of them are among the most abundant birds in Germany [29]. In turn, larger body size may be associated with the prevalence of CS data because larger birds are reported significantly more often [30]. Thus, when participants do not use a complete checklist, where all species seen and heard are counted, but instead use presence-only (or ad libitum) reporting, on average participants report larger bird species out of proportion to their rate of observation. Correspondingly, CS data are overrepresented near footpaths and wetlands [31], meaning that the different habitats influence the CS data. It thus becomes clear that species selection should not be based on population size or CS data solely, as this creates a bias regarding the size and kinship or habitat of species. Additionally, some studies on knowledge about species based their selection on garden bird counts. Basing studies mainly on garden bird count data [7,26] can bias the species selection towards songbirds, diurnal species, and urban birds (garden birds), and this usually excludes well-known orders such as duck or owl species (simply because ducks and owls do not usually visit feeders). By focusing only on garden birds, an advantage for garden owners is also created as they are more likely to know the species than others due to frequent sightings [32]. More specifically, people who own a garden may score higher in their initial basic knowledge about bird species [24]. Hence, covering several habitats is recommended, to give people who spend more time in forests or urban parks than in the garden a chance to show their knowledge. This does not mean that previous studies have been flawed in any way, but rather that the species serving as a baseline for knowledge about species should be well chosen and that more criteria should be used for species selection to reflect the diversity of birds.

## 2. Aim of the Study

**Figure 1:**
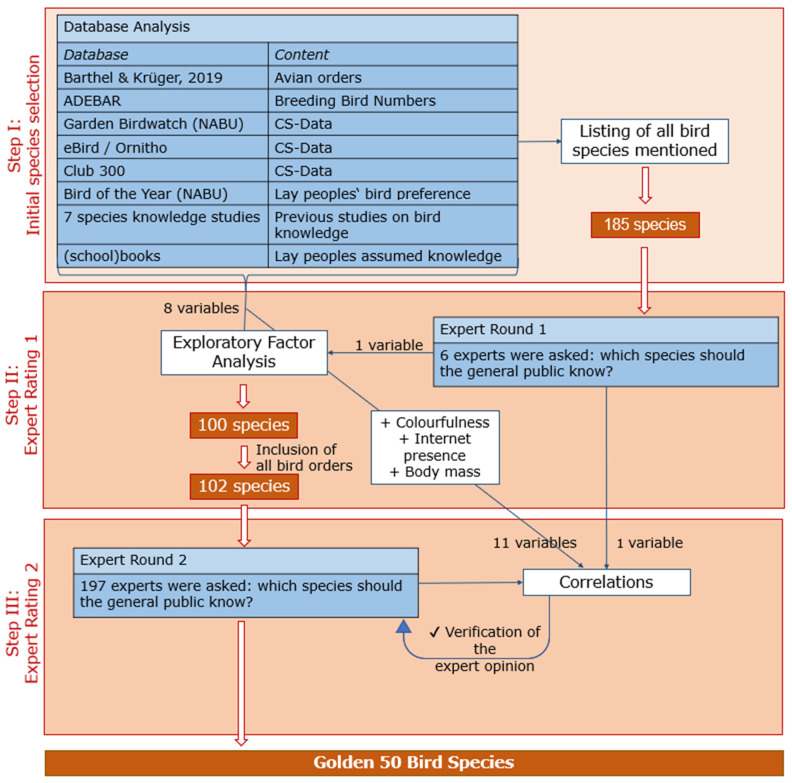
Overview of the methodological approach, divided into Step I (upper box), Step II (middle box), and Step III (lower box). The blue boxes describe what was done in each step of the selection, the white boxes describe the associated statistical approaches. The red boxes refer to the number of species as a result of each step.

The aim of this study is therefore to provide a list of the ‘golden 50′ bird species that is considered as a baseline for knowledge about bird species (see Step III for the reasoning behind the number of species). Here, for the first time, we present a selection procedure based on a variety of databases and two expert panel studies. The selection process applies to German species but can be transferred to any other biogeographical region and even to other taxa. Thus, it can be considered a template for further studies carried out in a similar manner elsewhere in the world. We analyzed bird-related databases such as CS online platforms (Club 300, eBird, Ornitho, Garden Birdwatch (NABU)), breeding bird data (Breeding Bird Census (ADEBAR), as well as bird-related books, previous studies, and the Bird of the Year election (NABU). We, therefore, acknowledge both the scientific basis of birds and their representation in society, and we add the human dimension to the selection procedure. The methods of this paper are based on three studies: Step I: Data Analysis, Step II: Expert Rating 1, and Step III: Expert Rating 2. An overview of the methodological approach is shown in Figure 1.

## 3. Material and Methods

### 3.1. Step I: Initial Data Analysis for the Expert Rating

In order to establish a general baseline of common and possibly known bird species, several bird-related databases were consulted according to certain criteria. The data mostly relate to population sizes, CS platforms, the popularity of bird species, previous studies on knowledge about species, and birds in the literature. A more detailed description of the databases and criteria used is given below. Only bird species included in the official German bird list [33] were used. Since 1800, 527 bird species have been reported in Germany, of which 250 breed here [33].

**1. Avian orders**: We used at least one species from each avian order reported in Germany [33]; however, 5 orders were excluded due to their rare occurrence or extremely localized distribution (see Table 1 for details). *Correction for Passeriformes:* As songbirds (*Passeriformes*) make up the largest proportion of extant bird species (more than 50% [34]), 86 songbird species were selected, with at least one species from each songbird family (with a few exceptions), to represent each family (Table 1).
**2. Breeding Bird Numbers**: The 118 most common breeding bird species were selected based on the German Breeding Bird Census ADEBAR [29]. Species with a population size larger than 10,000 (measured based on breeding pairs, territories, etc.) were used. Where a range was given, the lowest level was used. This selection is based on a nationwide breeding bird mapping, and it reflects the breeding avifauna. However, some species that fall within the range of the above criteria are largely unknown and poorly observed (e.g., the woodcock (*Scolopax rusticola*)) but were retained in our first round of selection. Depending on the population size, each species received the following rank: 1. >10,000; 2. >100,000; 3. >1 million.
**3. Citizen Science data—Garden Birdwatch**: CS data come in a variety of forms, from low-level engagement projects (counting garden birds) to more complex and advanced projects (e.g., ringing programs) [35]. The representation of bird species in the general population was used as an additional criterion. Here, we used the CS element of the Garden Birdwatch (“Stunde der Gartenvögel”), which requires only one hour of birdwatching and is open to everyone [7]. This event is run by the nature conservation organization (NGO) NABU, which has a strong base in Germany and in which the German birding scene has its roots. The species recorded during these days can be considered as species that the general public and less trained birders can encounter and identify daily. We used the 130 most common species from this event. The data are based on ranks, with rank 1 = the most commonly reported species.
**4. Citizen Science data—eBird and Ornitho**: More complex CS programs are the online platforms eBird (eBird.org) and Ornitho (ornitho.de). People who use these platforms tend to have higher knowledge and more skills than birders who do not use them [36]. Although these data are extremely useful for scientific analyses, they are slightly biased towards species near wetlands, species near footpaths [31], and larger species [30]. These data reflect the bird species encountered and reported by experienced observers, including summering and wintering species, but also migrants. The use of data from these platforms ensured that non-breeding species were included. The 100 species most commonly reported in Germany from eBird and 50 from Ornitho, extracted on 16 July 2022, at 12:10, were used (data is freely available on the platform). Data were ranked, with rank 1 = the most commonly reported species.
**5. Citizen Science data—Club 300**: Another association providing a platform for birdwatchers is the Club 300 Germany [37]. The platform has a membership fee of only 15 euros per year, which means that no one is excluded from the platform by their financial situation. This platform aims to improve the rapid transmission of information on the occurrence of rare bird species. Club 300 comprises the most knowledgeable and highly specialized birders in Germany [37,38]. However, the requirement to have seen at least 300 bird species in Germany to be admitted to Club 300 has been lifted for a while now. Consequently, there are also members with less than 100 species on the life list in Club 300 [37]. We used the Club List, i.e., the list of all bird species seen in Germany by at least one member of Club 300. We then selected the 100 species that have been seen by the highest number of Club 300 members, extracted on 16 July 2022, at 12:49 (data is freely available on the platform). Based on the number of observers of a species, a rank (1–18) was assigned to it, whereby rank 1 = highest number of observers.
**6. Lay peoples’ bird preference**: In 2021, the NGO NABU (see above) launched a unique challenge to select the most popular bird species in Germany. Based on this selection, the annual election of the Bird of the Year was then organized. Normally, the choice is made by a small commission of experts or by the general public on the basis of a preselection. In 2021, any bird species could be nominated and lay people as well as members of NABU were invited to take part. This vote can be seen as a representation of the status of a species in the eyes of the public and provides an alternative view in addition to the scientific census methods and CS data. Here, we have used the first 50 species selected on the basis of their rank, with rank 1 = the most popular species.
**7. Previous studies on bird knowledge**: As previous studies have already addressed bird species and public knowledge, we added the species that have been already used by Gerl, Randler and Neuhaus [6], Randler [9], Randler and Heil [24], Sturm, Voigt-Heucke, Mortega and Moormann [22], Cox and Gaston [10], Hooykaas, Schilthuizen, Aten, Hemelaar, Albers and Smeets [7], Jaun-Holderegger [39], Wolff and Skarstein [40], and Skarstein and Skarstein [41] to the list. This list comprises all available studies covering the topic in Europe published with a presentation of the respective selected species. Data from Randler [9] and Randler and Heil [24] as well as from Wolff and Skarstein [40] and Skarstein and Skarstein [41] were combined because these data were from the same research groups, so each research group could only contribute once to the dataset. Data were based on the number of research groups in whose studies a respective species was used (coded from 0–7, where 0 = used by no research group and 7 = used by 7 research groups).
**8. Lay peoples’ assumed knowledge**: As a supplement, we used a popular German public book “100 species everyone should know” [42], which covers a wide range of vertebrates and invertebrates; a basic bird identification book (covering 85 bird species; Haag [43]), and three school textbooks from grades 5 and 6 [44,45,46], as birds are especially mentioned in the curricula for these grades. In addition, we used a school textbook from 1933 [47]. This was done to consider what was taught in school at this time, as knowledge about species is often transferred across generations. We analyzed all books for the bird species they contained. The data were based on the number of mentions (coded from 0–6, with 6 = mentioned in all books).

The first step was to search the databases for all species that met the criteria described above. All species that fulfilled the criteria were collated in the first round. To check whether our selection reflects avian diversity, we compared the number of species per order reported in Germany [33] with the number of species per order on our list. For this, a chi-square test was performed in SPSS 28 to see if the number of species per order differs between the entire German list and our reduced list. This can be seen as a test of whether the selection in the list remains representative.

### 3.2. Step II: Expert Rating 1

The initial list contained 185 species and was presented to a small group of experts (n = 6) in a second step. The experts were asked for their opinion on which species should be known by the general public (in Germany). With this question, we wanted to obtain an individual assessment of the experts, almost as a supplement to the data analysis. These experts were chosen because they were all members of Christoph Randler’s Lab, and thus, were ornithologically versed and birdwatchers. The instruction was to mark at least one species per order, and as the order *Passeriformes* provided the most species of the initial selection, the experts were asked to mark one species per family in this order. In total, a maximum of one hundred species could be selected by each expert, but it was also possible to mark a smaller number of species. Each expert worked alone. The expert panel consisted of 2 males and 4 females, either experts in geoecology/ecology/ornithology (2 B.Sc., 1 M.Sc.) or biology education (2 M.Ed. in formal, 1 M.Ed. in non-formal education). These individuals were informed that their expert help would be needed to prepare the subsequent larger expert assessment. The experts in this first initial panel have experience with birds and birding in all parts and federal states of Germany, covering eastern, western, northern, and southern Germany, including some months or years of working/living in these different regions. In addition, students from Christoph Randler’s field ornithology course were asked to make similar assessments at the end of the summer term, first individually and then in small groups (total n = 26). The students had learned about bird identification from different lecturers since the beginning of their studies and had some experience in teaching school children as pre-service teachers. We followed the approach of the wisdom of the crowd [48] and aggregated the data from the 26 students into one single score. Hence, they were treated as an additional expert to reflect the students’ opinions and not outweigh the other experts. In total, therefore, seven expert assessments were used in this step (six people plus one aggregated score from the students). Each species could then receive a score between 0 (not mentioned at all) and 7 (mentioned by all). This expert assessment was then included as a dimension in the exploratory factor analysis (EFA). In this way, the social expertise was already added to a small extent in the first reduction of species.

SPSS 28 was used for the statistical analysis. To reduce the initial selection of 185 species to around 100 for the second panel of experts, we applied a factor analysis to the species and their characteristics. EFA helps to produce a more practical dataset by reducing the number of variables. More specifically, the manifest variables should be reduced to a small set of latent variables that explain as much of the variance in the outcome variables as possible. The starting point of EFA is an orthogonal decomposition of an input matrix. This results in an output matrix made up of orthogonal components (factors) that maximize the variation in the manifest variables. Thus, the EFA almost always results in a smaller number of factors [49]. In this way, the EFA was used to reduce the number of bird species.

For the EFA, we used the eight data sources of Step I (eBird, Ornitho, Club 300, Garden Birdwatch (NABU), Breeding Bird Census (ADEBAR), previous studies, books, Bird of the Year (NABU)) and the expert assessment as well. More specifically, we used the ranks, categories, and numbers already described above and included them in the factor analysis. Typically, EFAs overestimate the number of factors to be extracted when the decision is based on the eigenvalue greater than one criterion. To get a better estimate of the number of factors to be extracted, we performed a parallel analysis [50]. This parallel analysis helps to decide how many factors to extract and generates a random dataset and random eigenvalues. These random values are then compared with the values obtained from the exploratory factor analysis. The number of the factors to be extracted is the number for which the eigenfactors of the EFA are higher than the eigenfactors of the parallel analysis [50]. We used a principal component exploratory factor analysis with varimax rotation. In addition, we repeated the EFA without the data from expert round 1 to assess whether experts are needed to establish a species selection. To compare the ranks resulting from EFA 1 and EFA 2, a Wilcoxon signed-rank test was performed. After Step I, a chi-square test was calculated to test whether the reduced list still represents the avian diversity in Germany.

### 3.3. Step III: Expert Round 2

A second expert assessment was used for a further reduction to 50 species. It followed the wisdom of the crowd approach [48], i.e., the idea that a given number of expert opinions represents the ‘true’ value. We did two analyses in this expert round. First, a quality check of our experts, and second, the reduction of the species set. This reduction was the main aim of expert round 2, but nevertheless, we present some basic demographic data and validity on our experts.

For the expert assessment, people throughout Germany were contacted who generally have a high level of ornithological knowledge (due to their profession or leisure activities). These included students, postgraduates, or university staff in biological disciplines, members of nature conservation organizations or ornithological working groups and associations, employees of nature conservation authorities, and experts from ornithological internet platforms.

For this second expert panel, the reduced list of bird species from expert panel 1 was presented in an anonymous online survey. The experts were asked whether they thought the following bird species should be known to the general public (in Germany). The response options were either yes, probably, or no. Each “yes” was coded as 1, each “no” as −1, and each “probably” as 0. In addition, the experts were allowed to indicate other species that they considered relevant. After rating the species, the participants were also asked to provide some information about themselves (gender, age, highest level of education) or their qualifications as an expert (e.g., whether they are a teacher, members of a nature conservation organization). In addition, participants’ birding specialization was assessed using a 5-item scale c.f. [24]. Birding specialization refers to the knowledge of bird species and is self-reported. Participants had to indicate the number of species they could identify by sight or by song without any help, give a self-assessment of their ornithological skills on a scale from 1 = novice to 5 = expert, and answer two questions on behavior, i.e., the number of field trips and the number of days out. Previous work has shown that self-reporting is a highly reliable measure of knowledge, and the self-assessment of birding specialization was highly correlated with a subsequent knowledge test (r = 0.729, *p* < 0.001; Randler and Heil [24], see also Rögele et al. [51]). Thus, respondents can accurately assess their knowledge. The number of 50 species was chosen because we felt it reflects bird diversity in Germany well. So far, a maximum of 28 species have been surveyed in studies on knowledge (Randler and Heil [24]), but in order to not neglect people who know more than 28 bird species, a higher number of species is recommended [36]. To not scare people off, the questionnaire should have a comfortable length, which is why we see 50 species as a good number.

#### Testing the Suitability of the Expert Round 2

A correlation was calculated to examine the extent to which the assessment of the second expert panel was related to the variables of the initial preselection. For this purpose, some information derived from the databases was subject to more detailed assessment. This applies to the eBird, Ornitho, Club 300, the NABU, and the ADEBAR databases. For eBird and Ornitho, the exact number of reported observations was now used. Furthermore, each of the species was assigned a category based on the number of observations made by Club 300 members (on 16 July 2022). Depending on how many observers a species had, it was assigned a category. Four species had the most observers overall and thus received category 1 (547 observers). The Eurasian Nightjar (*Caprimulgus europaeus*) received category 81 with the smallest number of observers (440 observers). Each species was also ranked according to its position in the 2021 Bird of the Year and the Garden Birdwatch 2022 election of the NGO NABU. For the number of breeding pairs in Germany, the estimated number, according to the ADEBAR [29], was used for each species in the second round. In addition to the databases from the first round, further variables were correlated with the expert assessment 2: data on the colourfulness c.f. [25], the body mass data from [52], and the internet presence of the bird species, as well as the result of the expert assessment 1. The colourfulness of a species was assessed following the criteria of Randler, Staller, Kalb and Tryjanowski [25], who showed that the assessment of colourfulness by a few experts correlates with the opinion of the general public. Consequently, a colourfulness index was created for each bird species on the list (number between 1 = very dull to 5 = very striking). To determine the web presence of a species, the number of web pages it is mentioned in was assessed using an automated and depersonalized web search (using the Google Custom Search API). For this purpose, German species names were used and the Google hits were restricted to Germany. Synonymous species names were also used, and the number of hits was added. It was expected that the web presence would give a value reflecting the importance of a species to humans. The German names in this study follow Barthel and Krüger [33], and the English names were extracted from eBird.org. These correlations aimed to find out whether there is a relationship between all the above-mentioned variables, which reflect the popularity and abundance of bird species in society, and the expert assessment. It also allowed us to test whether an expert assessment is a suitable tool for compiling a species list. SPSS 28 was used for the statistical analysis. We used non-parametric tests (Spearman-Rho) to assess relationships and t-tests to compare groups. As after Step I and II, a chi-square test was calculated to test whether the reduced list still represents the avian diversity in Germany.

## 4. Results

### 4.1. Step I: Initial Data Analysis for the Expert Rating

The data analysis based on the above-mentioned criteria resulted in an initial list of 185 bird species (see Supplementary Materials S1). This was achieved by adding up all the bird species that have been identified in the process. To be included in our initial list, a bird species had to be mentioned in at least one of our eight categories from above. Because this initial selection includes scientific approaches (e.g., breeding bird mapping), CS data, general public views, human dimensions, and more societal approaches, many topics dealing with birds have been considered. This selection was used to inform the second step of the reduction, the expert assessments. The avian diversity in Germany was equally represented in the selection (χ^2^ = 29.100, df = 24, *p* = 0.216), and the selection can be considered representative in the numbers of species per order.

### 4.2. Step II: Expert Round 1

The EFA 1 (including the expert opinion) showed eigenvalues of 4.705 and 1.227, while the random eigenvalues were 1.346 and 1.221, suggesting a single-factor solution. When applying the EFA 2 without the expert opinion, eigenvalues were 4.079 and 1.221 (for all factor loadings, see Table 2). In EFA 1 and EFA 2, the loadings were below the usual threshold (<0.45, see Comrey and Lee [53]) for Bird of the Year and Club 300 members. Nevertheless, we kept the data from these platforms in the analyses because they were based on a positive preselection. Based on this analysis, the standardized residuals were saved and ranked. High values of the standardized residuals (z-scores) indicate a high level of agreement between the species characteristics and the experts’ responses (in case of EFA 1). More specifically, species with high z-scores occur frequently, are often reported on CS platforms, are liked, are well represented in literature/other studies, and were considered important to know by Round 1 experts. EFA 1 thus helps to reduce the number of species based on the criteria from Step I and the first expert panel. Comparing the ranks of EFA 1 with the ranks of EFA 2 (see Supplementary Materials S2), it becomes clear that the first 27 species were identically ranked and a further 3 species had the same ranking. Consequently, 30 species have identical rankings in the lists, and the remaining species mostly differ only slightly in their ranking. The Wilcoxon signed-rank test showed that the ranks resulting from the two EFA were not significantly different (z = −0.327, *p* = 0.743). Moreover, the correlation between both factor scores (standardized residuals) was extremely high (r = 0.993, *p* < 0.001, n = 185). Thus, it seems that the expert rating did not contribute much to the species selection.

The first 100 species with the highest residuals (z-scores) from EFA 1 were selected for the second expert panel. This resulted in the exclusion of one order (*Caprimulgiformes*) and one songbird family (*Bombycillidae*) which were not among the first 100 species. *Caprimulgiformes* and *Bombycillidae* each contain only one species occurring in Germany. The Eurasian Nightjar (*Caprimulgus europaeus*) is nocturnal, and the Bohemian Waxwing (*Bombycilla garrulus*) is an irregular/irruptive winter visitor. To include all orders and songbird families, they were added back to the species selection, resulting in 102 species being presented to the second round of experts. These species are listed in descending order in Supplementary Materials S2. We compared the number of species per avian order in Germany [33] against our selection of 102 species. The selection was representative (χ^2^ = 36.107, df = 24, *p* = 0.054).

### 4.3. Step III: Expert Round 2

#### 4.3.1. Analysis of the Expert Responses

**Figure 2:**
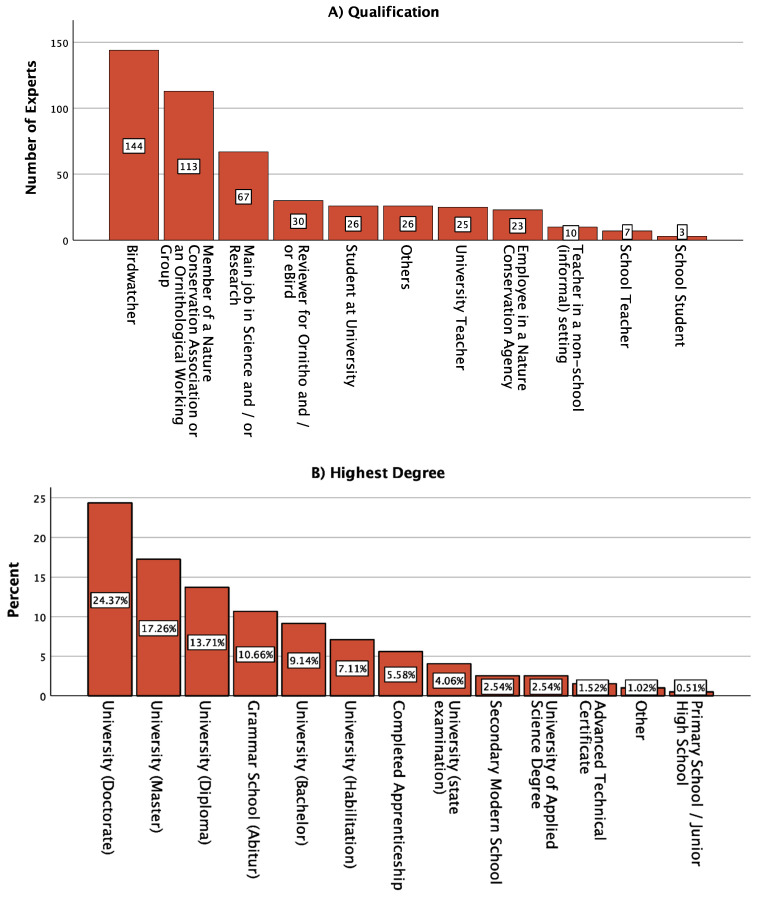

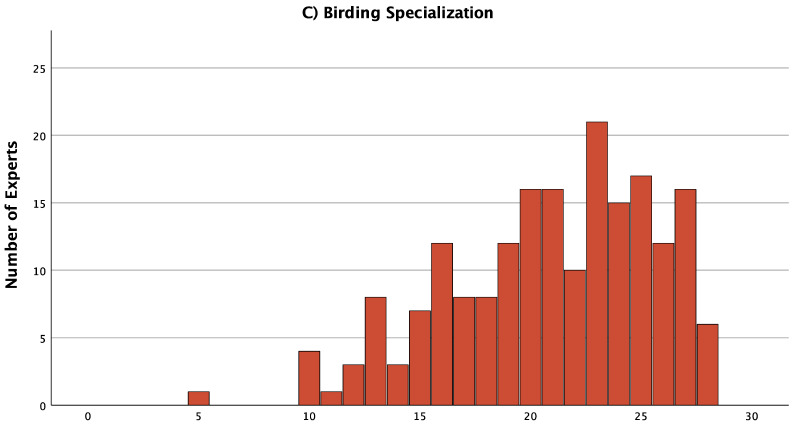
Analysis of the Experts of Step III: (**A**) Qualification as an Expert (multiple responses were possible) (**B**) Highest degrees of the Experts (in percent) (**C**) Histogram of the Birding Specialization of the Experts (5 Items; Maximum: 28 points (highly specialized), Minimum: 5 points (little specialized); Mean: 20.91 ± 4.66.

In Step III, 197 experts took part in the online survey. The average age was 44.42 ± 16.23 years, with 73 females (37.1%), 123 males (62.4%), and 1 person who did not specify gender (0.5%). Participants were asked to indicate their qualification as an expert, e.g., birdwatcher or other. Multiple responses were possible. Most of the respondents reported that they were birdwatchers, followed by members of a nature conservation association or an ornithological working group (Figure 2A). The most commonly reported highest degree was a university doctorate. This was followed by a master’s degree and diploma (Figure 2B). Birding specialization was 20.91 ± 4.66 (out of a maximum of 28 and a minimum of 5 points, Figure 2C). There was no correlation between the birding specialization score and the number of species selected, suggesting that more skilled people are aware of the difficulties of bird identification. Age was significantly negatively correlated with the number of species selected (r = 0.204, *p* = 0.004). Older respondents selected fewer species. Females selected a similar number of species as males (T = 1.56, *p* = 0.12).

#### 4.3.2. Analysis of the Species

**Figure 3:**
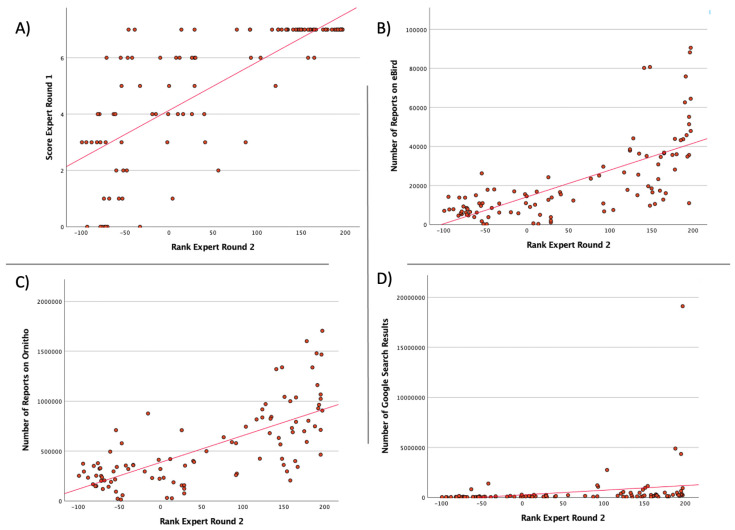
Correlations (Spearman-Rho) of Expert Round 2 with: (**A**) Expert Round 1; (**B**) Number of observations (of the respective species) reported on eBird; (**C**) Number of observations (of the respective species) reported on Ornitho; (**D**) Number of Google search results.

On average, the experts selected 57.88 ± 13.27 species that should be known by the general public. The coding of the response options allowed us to calculate a rank for each bird species, potentially ranging from −197 (no expert thinks that people should know this species) to 197 (every expert thinks people should know this species). The species rankings are shown in Table 3. To compare the rank of the ‘golden 50′ species with the ranks from Step II, see Supplementary Materials S2. However, Club 300 and body mass data were moderately positively correlated, and all other variables were strongly correlated with the expert assessment (Table 4). All correlations were significant except for colourfulness (Table 4). This means that the colourfulness of a species does not seem to influence the experts’ assessment. The experts mainly voted for species that are frequently reported on CS platforms, such as eBird and Ornitho (Figure 3). They also considered species that are frequently googled as important to know (Figure 3). The actual breeding bird population in Germany (ADEBAR) also correlates significantly positively with the expert assessment. Species with a high number of breeding pairs were rated as important to know by the experts. It is also clear that even the experts of panel 1 were able to accurately assess whether a respective species should be known or not, as the correlation between the two expert assessments was high. The significant positive correlation of body mass shows a tendency of the experts to select larger species.

Based on the results, the second expert assessment can be used to determine the selection of species for studies on knowledge about species. Some experts also mentioned additional species with the White-tailed Eagle (*Haliaeetus albicilla*) being the most frequently mentioned with 11 nominations. With 11 “yes” votes, he would only reach 61st place and was therefore not included in the ‘golden 50′ selection. Considering these 50 species, the diversity in terms of species per avian order is representative of the German avifauna (χ^2^ = 22.856, df = 24, *p* = 0.528).

## 5. Discussion

In this study, we aimed to select species that serve as a baseline for knowledge about bird species in the general public, in particular the ‘golden 50′. However, our data allow any other numerical cut-off to adapt it to other studies and study aims. Further, the method depicted here can be used as a template and adapted for other taxa and biogeographical regions. The selection carried out in this study is the largest and most detailed decision-making process for a species selection to date. On the one hand, it refers to several databases and on the other, the expert survey is characterized by a very high number of participants, not only on a small group.

To achieve the goal of a species selection, we used a selection based on different criteria related to the bird species itself (e.g., abundance, presence in CS data, presence in literature) and two rounds of expert assessments. Through this three-step procedure, a species list was created that represents the bird diversity in Germany and can be used as a baseline for knowledge about species. This is of value for educational institutions and studies on knowledge about species to make them more comparable and to monitor changes in the future, comparable to bird populations monitoring. Most of these studies on knowledge aim to find out how knowledge about species can best be improved [21,24]. Since knowledge about species is an important basis for species conservation, the compiled list is a starting point for further research in this field.

Other studies on knowledge about species have frequently used some of the above categories for their species selection as well. For example, Zahner, Blaschke, Fehr, Herlein, Krause, Lang and Schwab [26] used NABU’s Garden Birdwatch to make a preselection for an expert panel. Consequently, garden birds were used in their study, as they were in Enzensberger, Schmid, Gerl and Zahner [21] or Cox and Gaston [10]. Sturm, Voigt-Heucke, Mortega and Moormann [22] selected species for their knowledge study based on common species encountered in Berlin, while Prokop and Rodák [54] used common species encountered in Slovakia. By using only isolated indicators or databases for species selection, bias in species selection can easily occur, as described in the introduction. Consequently, our species selection has the advantage of referring to multiple databases, not just garden bird counts or breeding bird data.

Nevertheless, our data analysis must also be viewed critically, as it also creates biases. As we wanted to represent bird diversity in the list, we compared the species per avian order of each selection with the number of species from the German avifauna [33]. We found that the avian orders were equally represented as the chi-square tests were all non-significant. In addition to this advantage over other studies, the most important difference to the lists already compiled by other studies is that we referred to different databases such as CS data (Club 300, eBird, Ornitho, Garden Birdwatch (NABU)), the Bird of the Year election (NABU), scientific studies (previous studies on bird knowledge, Breeding Bird Census (ADEBAR)), and bird-related literature. Hence, we generated as many independent prioritizations as possible that provided information on how common and possibly known different bird species are.

The data analysis provided a good starting point for the expert panel in Step II. By comparing the two EFAs in Step II, it became clear that expert opinion is not essential to further reduce the species list. Nevertheless, in this study, we decided to consider the expert opinion from Step II to add a social dimension to the study to differ from species selections already carried out. Through the three-step process, an effortful but well-founded list of species was created, which can now be applied throughout Germany. Moreover, the procedure can also be transferred to other biogeographical regions, but according to our results, the involvement of (so many) experts would not be necessary. Still, to achieve a reference to society, it is worthwhile to consult experts.

To provide the experts in Step III with a selection of bird species, the first expert assessment list was set at 100 species, respectively (to include all orders or songbird families) at 102 species. The significantly high correlation between expert assessment 1 with expert assessment 2 shows that it was useful to include a small panel of experts to narrow down the initial selection from 185 to 102 species. However, this also means that our small group of ornithologically experienced people was sufficient to assess which species should be known. Whether expert groups consisting of a few people are suitable for species selection would have to be verified by repeating the process several times.

This means that for previous studies on knowledge about species, such as those by Zahner, Blaschke, Fehr, Herlein, Krause, Lang and Schwab [26], which also involved a group of experts in the selection of species, a solid assessment of the test birds has probably already been made. As mentioned, however, selections were only made on the basis of garden birds. Furthermore, Jaun-Holderegger [39] used a small group of experts (two zoologists) to decide the perceptibility and abundance of the species. Involving experts in the species selection is certainly the right approach but, based on our results, it is recommended to undertake a data analysis of different databases in advance.

The experts of Step III appear to be representative of the German population in terms of age and gender distribution, as the average age in Germany is currently 44.6 years [55], and slightly more males than females pursue an ornithological leisure activity [56]. In addition, the self-reported birding specialization of the experts was, as expected, high. Almost three-quarters of the participants are active birdwatchers, so it would be expected that they would have a good knowledge of bird species. The participants covered a wide range of people involved with birds, both professionally and privately. This allowed the benefits of the wisdom of the crowd to be fully exploited [48]. This is also shown by the results of the correlation of the second expert assessment with all the data included in this study. This includes the databases of the analysis in Step I, the expert assessment 1 (Step II) as well as colourfulness, body mass, and Google search results. According to this, experts are good at estimating abundance and familiarity and intuitively include these in their choices when it comes to selecting a baseline for knowledge about bird species. However, the experts did not seem to base this on the colourfulness of the species, suggesting that they rely more on abundance and familiarity and do not prefer colourful species to dull species when asked this question. Because the subjective opinion of the experts was very much in line with the data analysis, the huge benefit of our expert assessments becomes visible: they allow us to strengthen the data analysis.

On average, the experts rated 57.88 species that should be known by the general public. This is close to the 50 species we had originally intended. The ‘golden 50′ list could be used as a guideline for future studies and adjustments are entirely possible if plausibly justified. Especially since past studies on knowledge about species used between 8 [57] and 28 [24] birds, but mostly between 12 and 16 [21,22,26,58,59], a reduction of the list for future studies on knowledge is worthwhile. The extent to which the species list is reduced then depends on the respective objective of the study. In this study, however, the list should not be reduced further, as it agrees with the number of species given by the experts. Comparing the ‘golden 50′ with the species lists of other studies, the used species are mostly included in our list. In the German studies by Sturm, Voigt-Heucke, Mortega and Moormann [22] and Gerl, Almer, Zahner and Neuhaus [59], only one species of each study is not included in our list (Hooded Crow (*Corvus cornix)* and Eurasian Siskin (*Spinus Spinus*)). However, all species of Randler and Heil [24] are represented. With a few exceptions, the species used in other European studies are also listed in the ‘golden 50′. In the Swiss study by Jaun-Holderegger [39], in the English study by Cox and Gaston [10], and in the Dutch study by Hooykaas, Schilthuizen, Aten, Hemelaar, Albers and Smeets [7], one species, two species, and three species are not represented respectively. This is probably mainly due to the slightly different species compositions in the countries.

The ‘golden 50′ list contained one neozoon, the Canada Goose (*Branta canadensis)*. This can be debated, as Canada Geese can have impacts on native bird populations [60]. Nevertheless, the Canada Goose appeared in all our databases used for species selection and was supported by our experts. If the Canada Goose is included in the species list, a discussion about non-native species and their impacts on ecosystems can be stimulated in an educational context, for example, opening up a new subject to be addressed. However, it can be discussed whether the White-tailed Eagle should be included in the list or not, as this species received the most support from the experts. Therefore, we would also be satisfied with a species selection that omits the Canada Goose and includes the White-tailed Eagle instead.

## 6. Conclusions

With the list of the ‘golden 50’ (Table 3), our work shows which bird species could serve as a baseline for knowledge about species. This list is therefore a template for the selection of species in studies on knowledge about species and enables future scientists working on knowledge about species to make an informed choice of species for their study. In addition, the list can be used by educational institutions to determine which species should be taught. The list can also help to select so-called flagship species. This all supports the promotion of knowledge about species, which seems immensely important in the context of species extinction. The selection process can be used again in the same way, to select species for studies on knowledge, for example, in other biogeographical regions or other taxa.

## Figures and Tables

**Table 1:** Overview of all bird orders and families, with the respective number of species in the initial selection (Step I), as well as the number of species after Step II and in the ‘golden 50′ species list. The reasoning for an order/family (for Passeriformes) being not included is also given.

Order	Families in this Order (Number of Species in the Initial Selection)	Reason If Not Included in the Initial Selection	Number of Species in Germany ^1^	Number of Species in the Initial Selection	Number of Species after Study II	Number of Species in the ‘Golden 50′
**Galliformes**	Phasianidae		8	5	2	1
**Anseriformes**	Anatidae		53	21	7	4
**Caprimulgiformes**	Caprimulgidae		2	1	1	
**Apodiformes**	Apodidae		5	1	1	1
**Otidiformes**	--	only found in three federal states; very low breeding population of 114 individuals		--		
**Cuculiformes**	Cuculidae		3	1	1	1
**Pterocliformes**	--	not breeding in Germany, no records in the last 10 years		--		
**Columbiformes**	Columbidae		7	5	4	2
**Gruiformes**	Rallidae Gruidae		13	4	3	2
**Podicipediformes**	Podicipedidae		6	3	2	1
**Phoenicopteriformes**	--	limited breeding range; naturalized population, less than 20 pairs		--		
**Charadriiformes**	Burhinidae Haematopodidae Recurvirostridae Charadriidae Scolopacidae Glareolidae Laridae Stercorariidae Alcidae		114	18	4	2
**Gaviiformes**	--	only rare winter visitors		--		
**Procellariiformes**	--	very restricted range to island of Heligoland		--		
**Ciconiiformes**	Ciconiidae		2	2	1	1
**Suliformes**	Sulidae Phalacrocoracidae		5	2	1	1
**Pelecaniformes**	Threskiornithidae Ardeidae Pelecanidae		13	3	2	1
**Accipitriformes**	Pandionidae Acciptridae		27	11	4	2
**Strigiformes**	Tytonidae Strigidae		12	6	2	
**Bucerotiformes**	Upupidae		1	1	1	
**Coraciiformes**	Coraciidae Alcedinidae Meropidae		4	2	1	1
**Piciformes**	Picidae		10	7	3	2
**Falconiformes**	Falconidae		9	4	1	1
**Psittaciformes**	Psittaculidae		1	2	1	
**Passeriformes**	Laniidae	Some families were excluded a priori because of extremely limited breeding ranges or low population size	8	1	1	
	Vireinidae		2	0		
	Oriolidae		1	1	1	
	Corvidae		10	7	7	5
	Bombycillidae		1	1	1	
	Paridae		7	6	5	2
	Remizidae		1	0		
	Panuridae		1	0		
	Alaudidae		9	3	1	1
	Hirundinidae		5	3	2	2
	Cettiidae		1	0		
	Aegithalidae		1	1	1	
	Phylloscopidae		15	3	2	1
	Acrocephalidae		13	6	1	
	Locustellidae		5	1		
	Sylviidae		12	4	4	1
	Regulidae		2	2	2	
	Troglodytidae		1	1	1	1
	Sittidae		1	1	1	1
	Tichodromidae		1	0		
	Certhiidae		2	2	1	
	Mimidae		1	0		
	Sturnidae		2	1	1	1
	Turdidae		18	5	4	2
	Muscicapidae		24	12	6	3
	Cinclidae		1	1	1	
	Passeridae		4	2	2	1
	Prunellidae		4	1	1	
	Motacillidae		15	6	3	1
	Fringillidae		20	11	9	4
	Calcariidae		2	0		
	Emberizidae		16	4	2	1
	Parulidae		2	0		

^1^ according to Barthel and Krüger [33].

**Table 2:** Factor loadings for step II. Column 2 lists the components of the EFA 1 with the experts, column 3 the components of the EFA 2 without experts. Negative loadings are found if ranks were used for the data source (because rank 1 was the most preferred/common species). The closer the loading is to the extreme value −1 or 1, the stronger the influence of the factor. The loadings can be interpreted as follows: > 0.71 (50% overlapping variance) = excellent, > 0.63 (40% overlapping variance) = very good, > 0.55 (30% overlapping variance) = good, > 0.45 (20% overlapping variance) = fair, and > 0.32 (10% overlapping variance) = poor [53].

Data Source	Component (EFA 1 with Experts)	Component (EFA 2 without Experts)
Garden Birdwatch (NABU)	−0.842	−0.842
eBird	−0.833	−0.843
Expert Round 1	0.825	
Books	0.822	0.814
Ornitho	−0.779	−0.778
Previous Studies	0.774	0.787
Breeding Bird Census (ADEBAR)	0.743	0.771
Bird of the Year (NABU)	−0.380	−0.362
Club 300	−0.208	−0.217

**Table 3:** Results of Step III: the ‘golden 50′ bird species as a baseline for knowledge about bird species. Answers of the experts were coded as follows: “Yes” = 1, “No” = −1, “probably” = 0 and the resulting sum was calculated for each species.

Rank	Species	Scientific Name	Sum
**1**	Common Magpie	*Pica pica*	197
**2**	Common Chaffinch	*Fringilla coelebs*	197
**3**	Eurasian Blackbird	*Turdus merula*	197
**4**	Great Tit	*Parus major*	196
**5**	White Stork	*Ciconia ciconia*	195
**6**	Eurasian Jay	*Garrulus glandarius*	195
**7**	Common Starling	*Sturnus vulgaris*	195
**8**	European Robin	*Erithacus rubecula*	195
**9**	Mute Swan	*Cygnus olor*	193
**10**	Great Spotted Woodpecker	*Dendrocopos major*	192
**11**	Eurasian Blue Tit	*Cyanistes caeruleus*	191
**12**	Mallard	*Anas platyrhynchos*	190
**13**	House Sparrow	*Passer domesticus*	188
**14**	Grey Heron	*Ardea cinerea*	185
**15**	White Wagtail	*Motacilla alba*	180
**16**	Barn Swallow	*Hirundo rustica*	178
**17**	Common Buzzard	*Buteo buteo*	178
**18**	European Goldfinch	*Carduelis carduelis*	175
**19**	Common House Martin	*Delichon urbicum*	167
**20**	Greylag Goose	*Anser anser*	165
**21**	Eurasian Wren	*Troglodytes troglodytes*	165
**22**	Eurasian Bullfinch	*Pyrrhula pyrrhula*	164
**23**	European Greenfinch	*Chloris chloris*	161
**24**	Eurasian Skylark	*Alauda arvensis*	160
**25**	Common Kestrel	*Falco tinnunculus*	158
**26**	Feral Pigeon	*Columba livia f. domestica*	158
**27**	Common Cuckoo	*Cuculus canorus*	154
**28**	Red Kite	*Milvus milvus*	151
**29**	Common Swift	*Apus apus*	150
**30**	Common Kingfisher	*Alcedo atthis*	148
**31**	Carrion Crow	*Corvus corone*	148
**32**	Black Redstart	*Phoenicurus ochruros*	146
**33**	Eurasian Nuthatch	*Sitta europaea*	144
**34**	Common Wood Pigeon	*Columba palumbus*	141
**35**	Eurasian Blackcap	*Sylvia atricapilla*	135
**36**	Yellowhammer	*Emberiza citrinella*	134
**37**	Northern Lapwing	*Vanellus vanellus*	133
**38**	Common Chiffchaff	*Phylloscopus collybita*	128
**39**	Great Cormorant	*Phalacrocorax carbo*	124
**40**	Eurasian Coot	*Fulica atra*	124
**41**	Eurasian Jackdaw	*Coleus monedula*	121
**42**	European Green Woodpecker	*Picus viridis*	117
**43**	Common Crane	*Grus grus*	104
**44**	Common Nightingale	*Luscinia megarhynchos*	93
**45**	Black-headed Gull	*Chroicocephalus ridibundus*	92
**46**	Common Pheasant	*Phasianus colchicus*	92
**47**	Song Thrush	*Turdus philomelos*	87
**48**	Great Crested Grebe	*Podiceps cristatus*	77
**49**	Common Raven	*Corvus corax*	56
**50**	Canada Goose	*Branta canadensis*	41

**Table 4:** Correlations (Spearman-Rho) of the variables with the rank of the Expert Round 2 (Step III).

	*r*	*p*
Expert Round 1	0.813	<0.001
eBird	0.736	<0.001
Ornitho	0.708	<0.001
Google Search Results	0.703	<0.001
Garden Birdwatch (NABU)	−0.700	<0.001
Previous Studies	0.667	<0.001
Bird of the Year (NABU)	−0.634	<0.001
Books	0.613	<0.001
Breeding Bird Census (ADEBAR)	0.325	<0.001
Club 300	0.269	0.006
Body Mass	0.207	0.037
Colourfulness	0.069	0.491

## Data Availability

The raw data supporting the conclusions of this article will be made available by the authors, without undue reservation.

## Supplementary Materials

The following supporting information can be downloaded at: https://www.mdpi.com/article/10.3390/ani13132230/s1.
